# Vaccination Behaviour Influences Self-Report of Influenza Vaccination Status: A Cross-Sectional Study among Health Care Workers

**DOI:** 10.1371/journal.pone.0039496

**Published:** 2012-07-11

**Authors:** Anna Llupià, Alberto L. García-Basteiro, Guillermo Mena, José Ríos, Joaquim Puig, José M. Bayas, Antoni Trilla

**Affiliations:** 1 Preventive Medicine and Epidemiology Unit, Barcelona Centre for International Health Research, Hospital Clínic, Universitat de Barcelona, Barcelona, Catalonia, Spain; 2 Clinical Pharmacology Service, Hospital Clínic de Barcelona, Barcelona, Catalonia, Spain; 3 Biostatistics Unit, Universitat Autònoma de Barcelona, Barcelona, Catalonia, Spain; 4 Department of Applied Mathematics I, Universitat Politècnica de Catalunya, Barcelona, Catalonia, Spain; Public Health Agency of Barcelona, Spain

## Abstract

**Background:**

Published influenza vaccination coverage in health care workers (HCW) are calculated using two sources: self-report and vaccination records. The objective of this study was to determine whether self-report is a good proxy for recorded vaccination in HCW, as the degree of the relationship is not known, and whether vaccine behaviour influences self-reporting.

**Methods:**

A cross-sectional study was conducted using a self-administered survey during September 2010. Considering the vaccination record as the gold standard of vaccination, the properties of self-report as a proxy of the record (sensitivity, specificity, positive predictive value, negative predictive value) were calculated. Concordance between the vaccination campaigns studied (2007–2010) was made using the Kappa index, and discordance was analyzed using McNemar’s test.

**Results:**

248 HCW responded. The 95% confidence intervals of coverage according to the vaccination record and to self-report overlapped, except for 2007, and the Kappa index showed a substantial concordance, except for 2007. McNemar’s test suggested that differences between discordant cases were not due to chance and it was found that the proportion of unvaccinated discordant cases was higher than that of vaccinated discordant cases.

**Conclusions:**

In our study population, self-reported influenza vaccination coverage in HCW in the previous two years is a good proxy of the vaccination record. However, vaccination behaviour influences the self-report and explains a trend to overestimate coverage in self-reporting compared to the vaccination record. The sources of coverage should be taken into account whenever comparisons are made.

## Introduction

Influenza vaccination in health care workers (HCW) is recommended to protect patients and workers alike. Measurement of the coverage achieved allows estimates of the protection obtained in all HCW, monitoring of trends between seasons and measurement of the impact of the intervention. Russell et al [1] stress the importance of using the same target population for spatiotemporal comparisons between coverage. When making comparisons, reported coverage numerators are acquired from two sources: a) self-reported surveys of HCW [2]–[4] and, b) vaccination records [5]–[8].

The relationship between these two forms of calculating coverage has been studied in at-risk populations in whom self-reported coverage seems a good proxy of coverage according to the vaccination record [9]. However, it is not known whether the same relationship exists in HCW. There are several arguments for considering self-reporting a good proxy for registered vaccination status, for example that HCW have no major reasons to confuse or forget vaccinations. HCW are otherwise healthy people and therefore undergo few diagnostic or therapeutic procedures which they are easily able to recall. However, self-report might not be a good proxy because influenza vaccination is recommended by most institutions in the world [10]–[12] and widely extended to health centres, therefore HCW might tend to declare they had been vaccinated. In addition, annual vaccination might induce errors if HCW do not get the vaccine every season.

Assessing the degree of association between self-report and vaccination records in HCW could help to improve the reporting of coverage and provide criteria to compare different centers and time periods. The main objective of this study was to determine whether self-report was a good proxy for recorded vaccination in HCW. To our knowledge, is the first time that this question is considered amongst HCW. Secondary objectives were to determine whether there was any recall bias according to HCW vaccination behavior, whether the time between vaccination and the report had an influence and which factors were related to concordance/discordance between the self-report and the vaccination record.

## Results

### Study Sample

**Figure 1:**
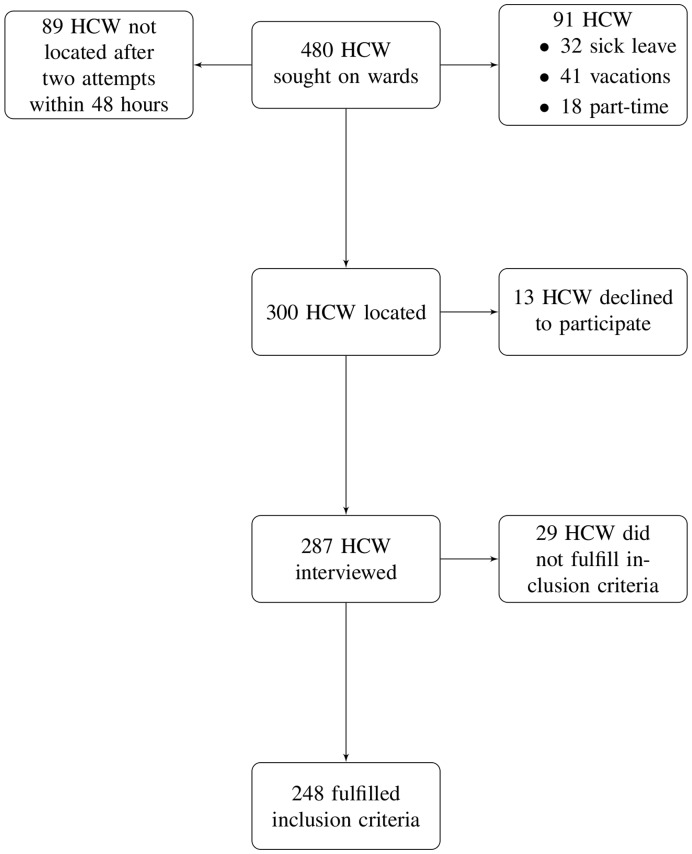
Flow chart for randomized HCW, participants, and subjects included in the study.

During the study enrollment period, 480 on-duty HCW were randomly selected (Figure 1). Of the 300 HCW located, 13 declined to participate (participation rate amongst located HCW: 95.7%). Finally, 248 HCW met the study inclusion criteria. The characteristics of the study sample are shown in Table 1. The sample population was mostly female (68.5%) and with a permanent contract (82.7%). Refusal to participate was similar in all professional categories.

**Table 1:** Characteristics of health care workers included in the study.

Characteristics	Study population (n = 248)	Overall population (n = 5258)
Female: n (%)	170 (68.5)	3725 (70.8)
Age in years: mean (SD)	46 (10.8)	44 (11.8)
Permanent contract: n (%)	205 (82.7)	3774 (71.8)
Seniority in years: media (SD)	17 (12.4)	14 (12.8)
Professional category: n (%)		
Staff physician	55 (22.2)	904 (17.2)
Resident physician	22 (8.9)	313 (6)
Nurse	53 (21.4)	1556 (29.6)
Auxiliary nurse/Orderly	34 (13.7)	943 (17.9)
Administrative/Technical staff	84 (33.9)	1541 (29.6)

### Self-reported Coverage as a Proxy for Coverage According to the Vaccination Record and Analysis of Discordance

Table 2 shows that, except for the 2007 season, self-reported coverage had a good concordance (95% confidence intervals (95%CI) and Kappa index) with coverage according to the Occupational Health (OH) vaccination record. In 2008, 2009 seasonal influenza (2009s) and A(H1N1)pdm09 influenza (2009A), self-reported coverage was a good proxy for coverage according to the OH vaccination record, although there was a trend to overestimate the coverage obtained through the OH vaccination record.

**Table 2:** Validity of self-reported influenza vaccination in HCW compared to occupational health medical records (2007–2009).

Campaign		Record		Coverage		Test characteristics						NVD/D (%)
		Yes	No	True	Self-reported	Sn	Sp	PPV	NPV	Kappa	McNemar	
				(95% CI)	(95% CI)	(95% CI)	(95% CI)	(95% CI)	(95% CI)	(95% CI)	P	
2007	**Declare Yes**	28	43	0.19	0.36	0.76	0.73	0.39	0.93	0.36	*p*<0.0001	82.7
	** No**	9	118	(0.14–0.24)	(0.29–0.43)	(0.60–0.87)	(0.66–0.80)	(0.29–0.51)	(0.87–0.96)	(0.21–0.51)		
2008	**Declare Yes**	52	18	0.33	0.33	0.73	0.87	0.74	0.87	0.61	*p* = 1	48.4
	** No**	19	122	(0.27–0.40)	(0.27–0.40)	(0.62–0.82)	(0.81–0.92)	(0.63–0.83)	(0.80–0.91)	(0.49–0.72)		
2009s	**Declare Yes**	71	25	0.32	0.39	0.91	0.85	0.74	0.95	0.72	*p* = 0.002	78.1
	** No**	7	145	(0.25–0.37)	(0.33–0.45)	(0.83–0.96)	(0.79–0.90)	(0.64–0.82)	(0.91–0.98)	(0.63–0.81)		
2009A	**Declare Yes**	28	13	0.13	0.17	0.90	0.94	0.68	0.99	0.74	*p* = 0.021	81.3
	** No**	3	204	(0.09–0.17)	(0.11–0.21)	(0.75–0.97)	(0.90–0.97)	(0.53–0.80)	(0.96–1)	(0.62–0.86)		

CI, Confidence Interval; PPV, Positive Predictive Value, NPV, Negative Predictive Value; Sn, Sensitivity; Sp, Specificity. NVD/D, Percentage of not vaccinated HCW who reported being vaccinated (NVD) over total discordant cases (D).

p<0.05, McNemar test.

Analysis of the characteristics of self-reporting for the four campaigns (table 2) shows that, considering the OH vaccination record as the gold standard, negative predictive values (NPV) were higher than positive predictive values (PPV). McNemar’s test showed that differences between discordant pairs did not appear to be due to chance. The direction of this difference between discordant pairs is shown in table 2, which calculates the proportion of discordant subjects for each vaccination status divided by the total number of discordant subjects. Except for the 2008 season, more discordant subjects reported being vaccinated when they were not than subjects who reported not being vaccinated when they were. In addition, the proportion of unvaccinated subjects who reported they were vaccinated was greater than the proportion of vaccinated subjects who reported they were not vaccinated. The 137 discordant subjects from all four campaigns studied were analyzed using the generalized estimating equations (GEE) model. No profile of vaccinated discordant subjects compared with unvaccinated discordant subjects was established according to sex, professional category, age, seniority in years and type of contract. Neither were differences found between unvaccinated discordant or concordant subjects and their self-report according to the same demographic variables.

## Discussion

The present study shows that self-reported influenza vaccination coverage in HCW is a good proxy for recorded vaccination coverage in the two previous years. The concordance, evaluated using the Kappa index and the CI of the coverage, supports this assertion. Self-reported coverage was consistently higher than coverage obtained through OH records in this study.

The finding that the proportion of unvaccinated discordant subjects was greater than the proportion of vaccinated discordant subjects may explain the tendency of self-reporting to overestimate coverage. Comparing these two groups in the different campaigns studied, in 2007, when the results show that self-reported vaccination was not a good proxy, the differences were even greater. This might suggest that either people remember better what they do than what they do not do or that, in case of doubt, people tend to be eager-to-please and thus state they are vaccinated. However, the underlying reason explaining these results needs to be further explored. With respect to the 2008 season, several factors may have influenced the fact that the coverage was not overestimated and that there was no disparity between discordant subjects. In 2008, a new model of vaccination campaign was introduced in our hospital [13], which may have better internalized by HCW, although there is not sufficient information to state that this occurred. In 2009, there were again a greater proportion of unvaccinated discordant subjects than vaccinated discordant subjects.

With respect to discordance in the self-report, taking the results of the 2009A campaign as an example, 20% of participants who self-reported being vaccinated were not, while 1.4% who had been vaccinated self-reported they had not. If it is assumed that the lack of memory is the same in vaccinated and unvaccinated subjects, the difference between these two percentages could be ascribed to being eager-to-please subjects who think they should have been vaccinated or that it is better to state they have been vaccinated because they recognize that vaccination is recommended by the hospital and the health authorities. Better characterization of these subjects could provide clues to approach groups who could potentially be convinced to be vaccinated, but the small sample of discordant subjects did not allow any pattern to be established. In the case of the 2009A campaign, contrary to what was expected given the low coverage achieved [14], no rejection effect was observed after A(H1N1)pdm09 influenza vaccination, when we expected to see vaccinated subjects who reported being unvaccinated. We also observed the eager-to-please effect, suggesting that HCW took no pride in not being vaccinated.

### Comparison with other Studies and Implications

The literature review found eight studies comparing self-report with vaccination record [9], [15]–[21]. These studies were conducted in high-risk patients in whom influenza vaccination was indicated and their results (Table 3) are aligned with the findings of this study which: self-reported coverage is a good proxy but tends to overestimate the coverage calculated from the vaccination record. McNemar’s test was calculated for all these studies and showed that discordance did not appear to be due to chance and that, of the total number of discordant subjects, the proportion of unvaccinated discordant subjects was greater than that of vaccinated discordant subjects. In addition, NPV were higher than PPV. Two studies [15], [19] retrospectively studied two vaccination seasons and found that self-report was a good proxy during these two seasons. High-risk patients exhibited the same behavior as our HCW, suggesting the presence of a social norm. In-depth studies of the qualitative aspects of the reasons leading HCW to be eager-to-please in order to define patterns would be of interest.

**Table 3:** Data of published studies comparing self-reported influenza vaccination in high-risk population.

First author,	Record			Coverage (95% CI)		Test characteristics						NVD/D (%)
Country, Year		Yes	No	True	Self-reported	Sn (%)	Sp (%)	PPV(%)	NPV(%)	Kappa (95%CI)	McNemar, p	
Hutchinson	**Declare Yes**	56	30	0.11	0.16	0.95	0.94	0.65	0.99	0.74	p<10^−6^	88.2
Canada, 1985	** No**	3	444	(0.08–0.14)	(0.13–0.19)					(0.65–0.82)		
Hutchinson	**Declare Yes**	143	15	0.29	0.30	0.92	0.96	0.91	0.97	0.88	p = 0.7	55.6
Canada, 1986	** No**	12	365	(0.25–0.33)	(0.26–0.33)					(0.83–0.92)		
Nichol	**Declare Yes**	32	10	0.37	0.47	0.97	0.82	0.76	0.98	0.75	p = 0.0117	90.9
USA, 1988	** No**	1	46	(0.27–0.47)	(0.37–0.58)					(0.61–0.89)		
Mc Donald	**Declare Yes**	94	21	0. 48	0.59	1.00	0.79	0.82	1.00	0.79	p<10^−6^	100.0
USA, 1999	** No**	0	80	(0.41–0.55)	(0.52–0.66)					(0.71–0.87)		
Zimmerman	**Declare Yes**	406	251	0.50	0.80	0.98	0.38	0.62	0.94	0.36	p<0.001	96.5
USA, 2003	** No**	9	153	(0.47–0.53)	(0.77–0.83)					(0.31–0.41)		
Andrews	**Declare Yes**	199	25	0.72	0.81	0.99	0.68	0.89	0.96	0.73	p<10^−5^	92.6
Australia, 1999	** No**	2	52	(0.67–0.78)	(0.76–0.85)					(0.64–0.83)		
Andrews	**Declare Yes**	212	14	0.78	0.81	0.97	0.77	0.94	0.89	0.78	p = 0.12	70.0
Australia, 2000	** No**	6	46	(0.73–0.83)	(0.76–0.86)					(0.68–0.87)		
Mangtani	**Declare Yes**	190	15	0.57	0.58	0.95	0.90	0.93	0.93	0.85	p = 0.56	57.7
UK, 2007	** No**	11	138	(0.52–0.62)	(0.53–0.63)					(0.75–0.95)		
Skull et al	**Declare Yes**	1309	180	0.77	0.86	0.98	0.56	0.88	0.91	0.62	p<10^−6^	89.1
Australia, 2007	** No**	22	226	(0.75–0.79)	(0.84–0.88)					(0.58–0.67)		
Irving	**Declare Yes**	1258	153	0.45	0.49	0.92	0.92	0.94	0.89	0.85	p<10^−6^	70.5
USA, 2009	** No**	64	1432	(0.43–0.47)	(0.47–0.51)					(0.83–0.87)		

CI, Confidence Interval; PPV, Positive Predictive Value; NPV, Negative Predictive Value; Sn, Sensitivity; Sp, Specificity. NVD/D, Percentage of not vaccinated subjects who reported being vaccinated (NVD) over total discordant cases (D).

p<0.05, McNemar test.

### Strengths and Limitations

One strength of this study is that the sample kept the proportions of professional category and vaccination coverage of the overall population in order to avoid participation bias due to unvaccinated subjects [15], [19]. The sample characteristics were consistent with those of the whole population of hospital workers (Table 1). In addition, the coverage of the sample calculated using the OH vaccination record matched the coverage of the total number of HCW recorded in the hospital, indicating that the sample was representative and consistent, given that the CI overlapped and collected the time trend well. Other strengths were that four vaccination campaigns were analyzed together and that the number of subjects who declined to participate was very low.

Studies [22], [23] have assessed the validity of vaccination records, which are also to some degree a proxy for real vaccination. It is difficult to confirm that a HCW is not vaccinated. However, we believe that our records are reliable. Most HCW can be presumed to be healthy and have few reasons for being vaccinated outside the workplace. In our hospital, the mobile unit covers all wards and is in contact with the majority of workers and also records the very-few HCW who report being vaccinated outside the hospital. A further limitation is that the Human Resources Department (HR) does not have a completely up-to-date record of the shift and location of each HCW, which would explain the number of workers not located or not known by their fellow-workers.

### Conclusions

In our study population, self-reported influenza vaccination coverage in HCW is a good proxy for recorded vaccination coverage in the two previous years. In the different campaigns studied in the present paper and in the previous studies, the results show that, of the total number of discordant subjects, the proportion of unvaccinated discordant subjects was greater than that of vaccinated ones, suggesting that vaccination behavior influences the self-report. This explains the tendency to overestimate coverage using self-report compared with vaccination records. The sources of vaccination coverage should be taken into account when comparisons are made.

## Methods

### Study Design and Population

A cross-sectional study was conducted in HCW of the Hospital Clínic of Barcelona (HCB), a 700-bed public teaching hospital, during a two-week period in mid-September 2010, as part of the PIVAC (Professionals and Influenza VACcination) project. In the framework of the PIVAC project, a survey was conducted before and after an influenza vaccination promotion campaign. This survey addressed various aspects related to influenza vaccination in HCW such as the determinants of influenza vaccination, motivations, risk perception of influenza for themselves, patients and relatives, the impact and acceptation of different strategies and the validity of self-declaration. A total of 480 HCW were sequentially sought during the recruitment period following a randomly sorted list of the HCW of the hospital. We aimed to recruit 420 HCW out of a population of 5258, stratified by professional category (5 groups) and vaccination behaviour in the previous season. Recruitment tables were updated daily by the four interviewers.

### Data Sources, Participants and Variables

We used three data sources: 1- PIVAC self-administered survey, 2- Vaccination history from OH medical records 3- HR datalist.

1- The items of the PIVAC survey used asked whether HCW had received influenza vaccination in the four previous campaigns (2007, 2008, 2009s and 2009A). 2- The OH medical record includes the act of vaccination and its date for each season. HCW in active work at the HCB in each season not registered in the OH vaccination record were considered as unvaccinated. Active HCW were considered as those working in the HCB for more than three, two and one years in the seasons 2007, 2008 and 2009, respectively. For each season, HCW surveyed who were not actively working in the HCB during this period were excluded.3- Demographic variables (sex, date of birth, years of work at HCB, contract type and professional category) were obtained from the HR datalist. The variable concordance was created by cross-matching the self-report with the OH vaccination record, with four categories: concordant vaccinated, concordant unvaccinated, discordant vaccinated and discordant unvaccinated.

### Vaccination Campaigns

Before 2008, influenza vaccination campaigns included educational-advertising material based on posters placed in strategic sites and institutional support at the onset by means of an e-mail to all HCW. HCW accessed free-of-charge voluntary influenza vaccination either by attending the Occupational Health Clinic, open from 8 a.m. to 17 p.m., five days a week, or by on-site contact with a mobile unit team staffed by one nurse per shift, including week-ends, who covered the whole hospital without previous forewarning of the route. Starting in 2008, a new model of campaign was implemented using incentives like prizes and the use of new technologies such as weekly emails and a hospital intranet website to post photos of vaccinated HCW [13].

### Statistical Analysis

The self-report was cross-matched with the OH vaccination records of the seasons included and with the demographic variables obtained. A descriptive analysis was made of the study population. For qualitative variables, the absolute frequency and percentage were determined. For quantitative variables, the central trend, position and dispersion were estimated by mean and standard deviation (SD). Taking the vaccination record as the gold standard for vaccination act, the basic properties of the self-report as a proxy of the vaccination record (sensitivity, specificity, positive predictive value, and negative predictive value) were calculated and their 95% Confidence Intervals (95%CI) was estimated. Concordance according to the vaccination campaign was analyzed using the Kappa index and their 95% CI [24] and discordance was analyzed using the McNemar test. The profile of discordant subjects was analyzed by means of a generalized estimating equations methodology using an unstructured matrix to account for within-subject variability. All analyses used a bilateral type I error of 5%. The analysis was performed using the SPSS v.15 and R v.2.12 statistical packages.

### Ethics

All study materials stated that the study was approved by the HCB Clinical Research Ethics Committee and that participation was voluntary. All personal data of HCW were dissociated and treated as confidential at all times.
